# The effects of icariine concentration on osteoclasts bone resorption induced by titanium particles *in vitro*

**DOI:** 10.1093/rb/rbv002

**Published:** 2015-08-10

**Authors:** Yiyuan Zhang, Yu Lin, Lili Xiao, Eryou Feng, Wulian Wang, Liqiong Lin

**Affiliations:** Joint Surgery Department, the Second Hospital of Fuzhou Affiliated to Xiamen University, Fuzhou 350007, China

**Keywords:** icariine, titanium particle, osteoclast, bone resorption, aseptic loosening

## Abstract

In artificial joint replacement, osteoclast bone resorption induced by wear debris of the implant is a main reason for aseptic loosening. To extend the life of the prosthesis, detailed mechanisms of aseptic loosening and the ways to prevent it should be explored. The aim of this study was to investigate the *in vitro* effect of icariine on the bone resorption of osteoclasts induced by titanium particles. Macrophage colony stimulating factor (M-CSF) and receptor activator of NF-kB ligand (RANKL) were used to generate osteoclasts from RAW264.7 precursors. The proliferation of RAW264.7 precursors in the presence of different doses of icariine was evaluated by MTT assay. The cells were treated with titanium particles, titanium particles with icariine and culture medium only (control), respectively. At 48 h after treatment, the expression level of receptor activator of NF-kB (RANK) was detected by ELISA, and messenger RNA (mRNA) levels of tartrate-resistant acid phosphatase (TRAP), matrix metalloproteinase 9 (MMP-9), carbonic anhydrase II (CAII) and Cathepsin K (CtsK) were determined by real-time polymerase chain reaction. Western blot was applied to analyze the expression levels of TRAP, RANK and CtsK. In addition, bone chips were cultured in the above conditions, and Toluidine blue staining was then employed to calculate the number and area of resorption pits in the bone chips. After treatment with icariine, expression level of RANK was significantly decreased in the RAW264.7 cell that induced by titanium particle and its cultural medium, mRNA and protein levels of TRAP, CAII, MMP-9 and CtsK were reduced as well. In addition, the numbers of bone resorption pits and areas on bone slices were both reduced by icariine challenging. Icariine could inhibit bone resorption of osteoclast induced by titanium particle, and it might be used as a promising drug for treating of aseptic loosening.

## Introduction

Artificial joint can reconstruct the joint function with lesions. However, with the extended of use of artificial joints, revision renovation gradually increased for various reasons. Many studies have focused on the mechanism and effective prevention of prosthetic loosening to extend the service life of prostheses. The main reason for aseptic loosening is wear particle-induced osteolysis [1]. In the use of artificial joint, wear debris is produced [2], which can elevate a series of inflammatory cytokines, leading to an increased osteoclast activity, bone resorption induction, osteoblast apoptosis induction and osteolysis acceleration [3, 4]. Titanium alloy is one of the most commonly used metal materials for manufacturing artificial joint, and the artificial joint made of titanium alloy also produce wear debris in clinical applications [5]. In the past, aseptic loosening was generally considered to be caused by wear debris produced from ultra high-molecular-weight polyethylene [6]; however, recent studies suggested that the metal wear debris is also an important reason for aseptic loosening of metal implants [7, 8]. Investigations have demonstrated that wear debris surrounding the prosthesis could loosen the prosthesis by activating the osteoclasts differentiation pathway, which can lead to bone resorption [9].

**Figure 1.**
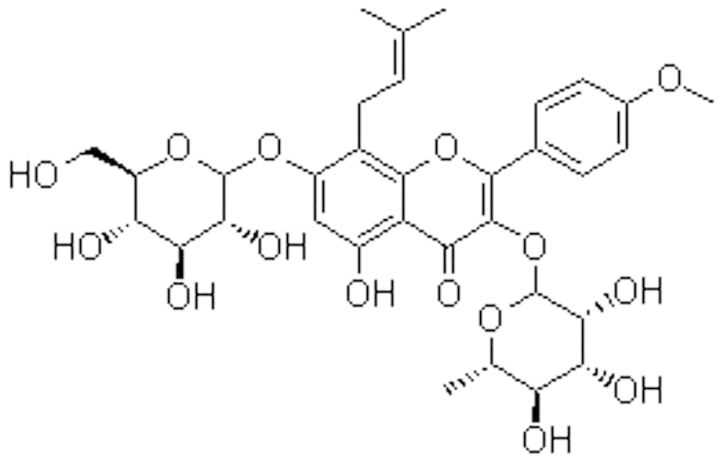
The molecular structure of icariine.

Natural estrogen, from traditional Chinese medicine, is eutherapeutic in inhibiting the osteoclast activity, osteoporosis, as well as increasing bone density [10, 11]. Icariine (C_33_H_40_O_15_, Fig. 1), which has been reported to have some estrogen activity, could inhibit proinflammatory interleukin-6 and tumor necrosis factor (TNF) expression and in turn, the bone resorption induced by them [12, 13]. However, the biological effect of icariine on *in vitro* titanium particle-induced osteoclast bone resorption has not been reported. In this study, we examined the potential *in vitro* titanium particle-induced icariine inhibitory activity on osteoclast differentiation, which might provide more understanding in terms of the underlying mechanisms and hence potential novel ideas for prevention of aseptic loosening.

## Materials and Methods

### Cell lines and compounds

RAW264.7 (Mouse leukemic monocyte macrophage cell line) was obtained from Guangzhou Jennio Biotech Co., Ltd. Icariine was purchased from Shaanxi Huike Plant Development Co., Ltd (purity: 98%, lot no: EP20070615).

### Main reagents and instruments

Macrophage colony stimulating factor (M-CSF) and receptor activator of NF-KB ligand (RANKL) factor were obtained from Sigma (St Louis, MO). Titanium particle was supplied by Nonferrous Metals Company (Beijing, China) with a mean diameter of 91 ± 15 μm. Trizol was purchased from Invitrogen (Carlsbad, CA). RANK ELISA kit was obtained from Xitang Biology Sci-tech Co., Ltd (Shanghai, China). The primers for tartrate-resistant acid phosphatase (TRAP), CA II, matrix metalloproteinase 9 (MMP-9), Cathepsin K (CtsK) and GAPDH were supplied by Sangon Biological Engineering Technology & Services Co., Ltd (Shanghai, China). The cDNA Synthesis and SYBR Premix Ex Taq polymerase chain reaction (PCR) Kits were purchased from TaKaRa (Shiga, Japan). The antibodies for TRAP, RANK, CtsK and β-actin were purchased from Cell Signaling Technology (Beverly, MA). GeneAmp PCR System 9600 was purchased from Perkin Elmer. The 7500 Real-Time PCR Systems was supplied by Applied Biosystems. Finally, the TRAP Staining Kit was obtained from Jian Cheng Bioengineering Institute (Nanjing, China).

### Preparation of titanium particles

The particles were baked at 300°C for 6 h, suspended in 25 ml of 75% alcohol/100 ml particles and vortexed for 24 h at 200 r/min. After centrifugation, the particles were suspended in 75% alcohol and were stirred for 24 h. They were then washed three times with PBS and were dried by UV curing. The cell toxicity was detected using Endotoxin Detection Kit, which showed that the concentration of endotoxin adhesion endotoxin was less than 0.10 EU/ml on particles (nontoxic to the cells) [14]. The particles were washed with a mixture of 25% nitric acid and 0.1mol/l NaOH several times. They were then resuspended in PBS to a final concentration of 5 wt% Ti and were autoclaved. A total of 4.5×10^7 ^particles were included in 1 ml of 0.1% (v/v) titanium particle suspensions. This solution was sonicated for 10 min before being applied for cell treatment to prevent adhesion.

### Preparation of bone chips

Fresh bovine cortical bone was used to obtain the bone chips with the size of 0.5 × 0.5 × 0.2 cm (L × W × T) using a wire saw and grindstone. They were sonicated for 10 min in distilled water, and the sonication was repeated three times. After being immersed in 75% alcohol for 24 h, the bone chips were air dried and treated on each side with 4 h of ultraviolet radiation, after which they were stored at −20°C.

### Osteoclast validation

The RAW264.7 cells were seeded into the plates overnight. They were then induced with the addition of a medium including 30 ng/ml M-CSF and 50 ng/ml RANKL. The cell growth morphology was observed under microscopy. After 6 days of incubation, the osteoclasts were verified by TRAP and toluidine blue staining of the bone resorption pits.

### Proliferative effects of icariine on RAW264.7

To validate the proliferation effect on RAW264.7 by different concentrations of icariine and to explore the optimum concentration of icariine, cell culture medium with different concentration of icariine was prepared by dissolving the icariine in dimethyl sulfoxide (Sigma-Aldrich Co. LLC.) and then diluted with DMEM containing 10% fetal bovine serum (FBS) to different concentrations (0.1, 0.5, 1, 5, 10, 15, and 20 mg/ml). The RAW264.7 cells were plated at a density of 1 × 10^4^ cells/ml in 96-well plates. After being cultured overnight, cells were treated with different concentration of icariine, eight wells for each concentration. After 72 h, 10 µl of alamar Blue was added and the plates were incubated in the dark for 4 h. The supernatant was collected and read at 590 and 560 nm using spectrophotometer.

### The challenge of icariine

The RAW264.7 cells were seeded into 6-well plates overnight, 30 ng/ml M-CSF and 50 ng/ml RANKL were added to the cells, followed by a 6-day culture. On the 7th day, cells were treated with icariine in the presence or absence of titanium particles, followed by a 48-h incubation. They were divided into three groups and cultured with different growth media as follows: control group: DMEM with 10% FBS; Ti particle group: 0.1vol% Ti particles + DMEM with 10% FBS; Ti + icariine group: 0.1vol% Ti particles + the optimum concentration of icariine + DMEM with 10% FBS.

### ELISA assay to detect the expression level of RANK

The medium was collected after 48 h incubation. Sample, sample diluent and standard sample were added to each well. After incubation for 30 min at 37°C, 50 μl of coupling fluid, substrate and termination agent were added into each well to determine the OD value at 450 nm.

### The mRNA level of TRAP, MMP-9, CAII and CtsK in osteoclasts

Cells were cultured for 48 h and the total RNA was extracted with TRIZOL. Total RNA from each sample was applied to agarose gels to determine the RNA purity and quantity by ethidium bromide staining, which was analyzed by gel imaging analysis system. The OD_260_ of the prepared total RNA was measured with spectrophotometer and then used to determine the RNA concentration. For cDNA synthesis, total RNA (500 ng) was used in each sample according to cDNA reverse transcription kits. The PCR amplification was performed in ABI7500 instrument, and primers for the genes are listed in Table 1. The threshold cycle (Ct) values were calculated, and amplification curves were obtained to evaluate the messenger RNA (mRNA) level of the genes. Each experiment was performed three times. The 7500 system SDS software was used for result analysis.

**Table 1.** The list of the primers

Gene name	Upstream primer	Downstream primer
GAPDH	CCGAGAATGGGAAGCTTGTC	AAGCACCAACGAGAGGAGAA
TRAP	CTCTGTGCGACATCAACGAAA	TTAGCGGACAAGCAGGACTC
MMP-9	CTCCGTGTCCTGTAAATCTGC	TCTGACCTGAACCATAACGCA
CAII	AGGGAGCCCATTACTAG	ATCCAAATCACCCAGCCGT
CtsK	TCGGAATAAGAACAACGCCTG	AAGCACCAACGAGAGGAGAA

### The protein level of TRAP, MMP-9, CAII and CtsK in osteoclasts

Cells were harvested for total protein extraction. Protein concentrations were determined by bicinchoninic acid method. A total of 30 μg of protein was loaded from each sample in 12% polyacrylamide gel. Proteins were electrophoretically separated and transferred to the PVDF membranes. For immunoblotting, the membranes were blocked with blocking buffer for 30 min. Antibodies were diluted 1:5000, and the membranes were incubated with them for 1 h. After being washed with 40 ml of washing buffer for six times (1, 20 and 5 min, twice for each), the blots were incubated with secondary horseradish peroxidase conjugated antibody for 30 min at room temperature. The blots were washed as before, the resulting immunoblots were visualized using ECL substrate. The quantification of protein expression was accomplished by using Phoretix 1D analysis software.

### Bone resorption pits were counted and the surface area was measured

The RAW264.7 cells were seeded into a 12-well plate, with one bone chip in each well. The medium with M-CSF and RANKL was added later and it was incubated for 6 days. On the 7th day, the medium containing titanium particles with or without icariine was added into the cells, while the medium alone was used as the control. After incubation for another 6 days, the bone chips were used for Alamar Blue staining analysis. The pits were counted in the whole piece of bone through a 100 × microscope, and the results were demonstrated as pit/bone. Bone resorption areas were counted from bone pit images by the image processing software.

### Statistical analysis

The results were expressed as mean ± standard deviation of these experiments. *F* test was performed for statistical analysis using the SPSS 13.0 package. Statistical significance was set at *P* < 0.05.

## Results

### Osteoclast induction and characterization

**Figure 2.**
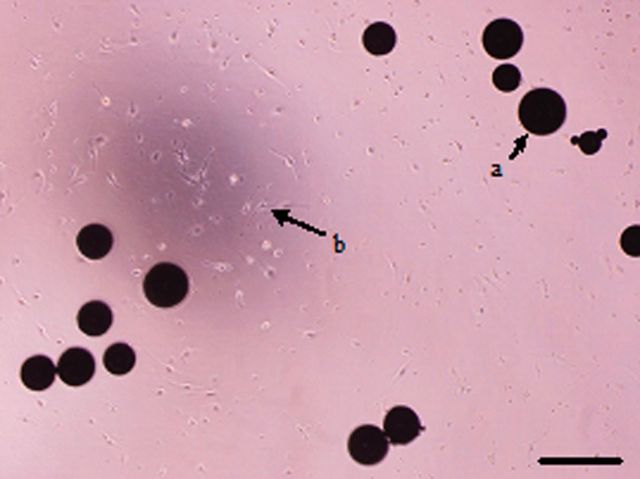
RAW264.7 cells co-cultured with Ti, bar = 100 μm (**a**: titanium particles, **b**: RAW264.7 cells).

**Figure 3.**
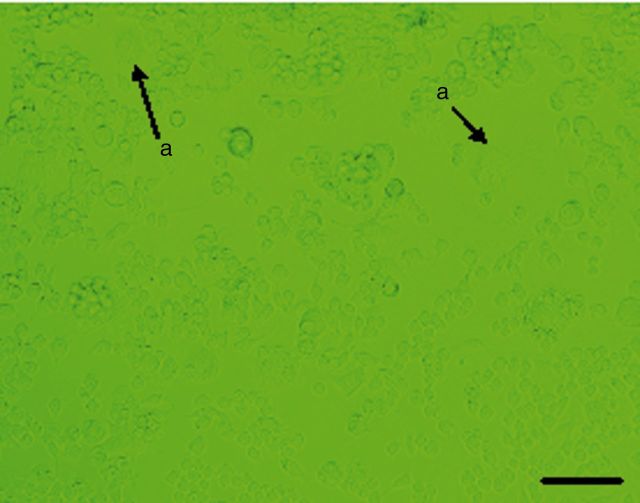
The RAW264.7 cells induced for 6 days, bar = 100 μm (**a**: osteoclasts nucleus).

**Figure 4.**
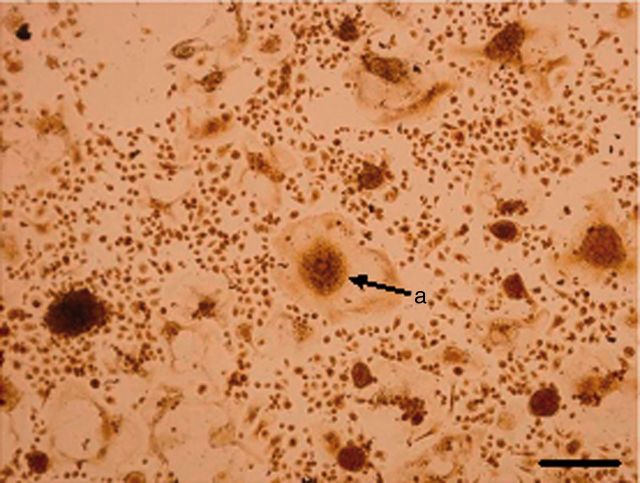
The TRAP staining, bar = 100 μm (**a**: TRAP staining osteoclasts).

**Figure 5.**
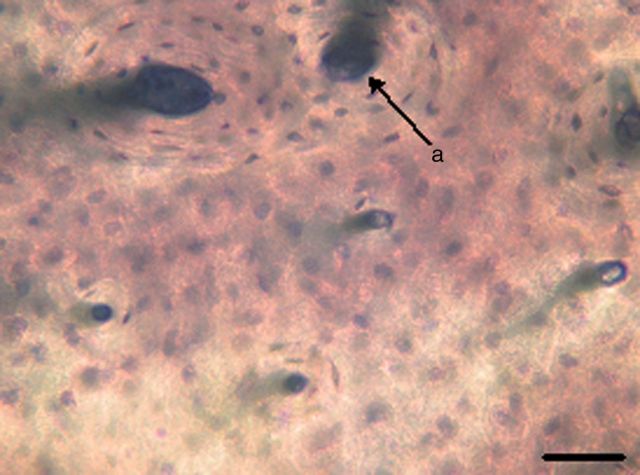
The toluidine blue staining, bar = 100 μm (**a**: absorption of lacuna bone resorption pits was counted and surface area was measured).

After induction for 4 days, the RAW264.7 cells were observed to begin differentiating (Figs. 2 and 3). The formation of large, multinucleated cells was observed with more pseudopods. Multinucleated cells with more than three nuclei appeared on the 5th day. On the 6th day, the number of multinuclear cells increased and more pseudopods appeared. The TRAP staining showed a reddish brown precipitation at the cytoplasmic area of the cells. They were negative for nuclear stain, and multinuclearity was observed (Fig. 4). A blue-purple ellipse and sausage-type staining were observed for bone resorption pits after toluidine blue staining (Fig. 5).

The bone chip was stained by toluidine blue, the bone resorption pit numbers were counted and the area was analyzed. It demonstrated that both the number and the area of the bone resorption pits were increased for the titanium particle treatment group when compared with that of the titanium particle and icariine treatment group (Table 2). However, the number and the area of the bone resorption pits were higher in both of these groups when compared with that of the control group (Table 2). There was a significant difference in the multiple comparison test among the three groups as presented in Table 2 (*P* < 0.05).

**Table 2.** The number and area of bone resorption pits in the bone chips in different groups

Group	Numbers of bone resorption pits*	Area of bone resorption pits (μm^2^)*
Control	21.32 ± 2.64	291.38 ± 36.58
Ti	33.84 ± 1.96	841.64 ± 63.26
Ti + icariine	27.64 ± 4.06	574.11 ± 36.35

*P < 0.05 in multiple comparison among the three groups.

### Icariine-induced RAW264.7 cell proliferation

**Figure 6.**
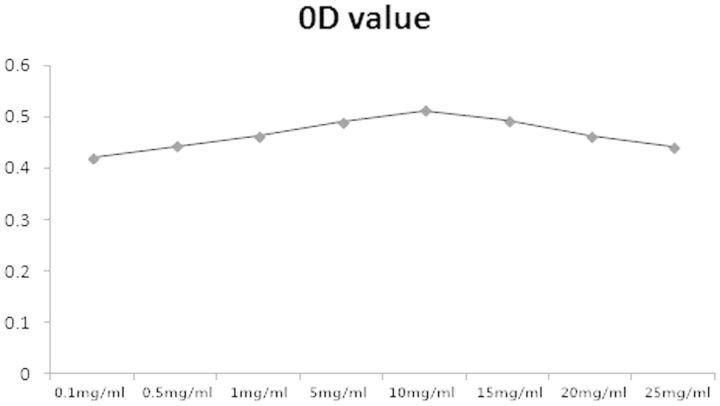
The effect of different concentrations of icariin on osteoclasts proliferation.

The MTT assay was used to determine the proliferation of RAW264.7 cells. It showed that Icariine improved the proliferation of RAW264.7 cells in a dose-dependent manner. Maximum improvement in cell proliferation was observed at a concentration of 10 mg/ml. Therefore, the concentration of 10 mg/ml of icariine was chosen for the rest of the studies (Fig. 6).

### RANK expression analysis

**Figure 7.**
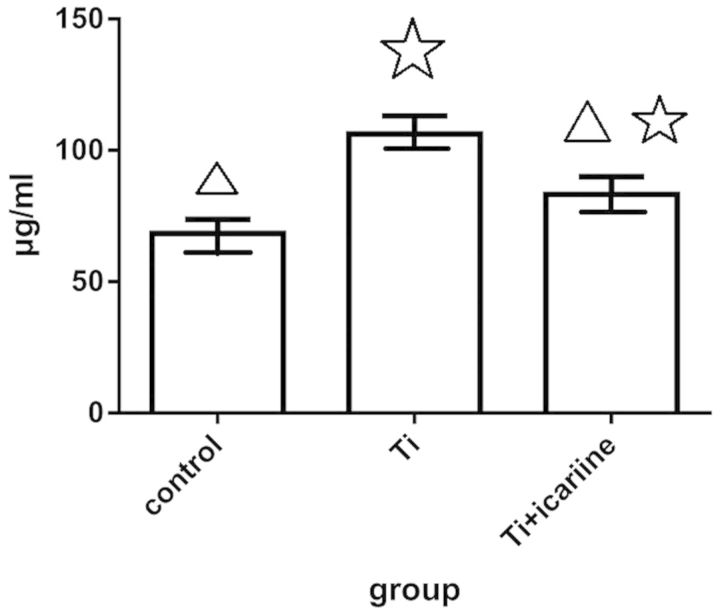
The expression level of RANK when compared with control (△: *P *< 0.01) and when compared with Ti, (: *P* < 0.01).

The levels of RANK expression in the cells were analyzed. The results showed that the expression level of RANK by Ti treatment was significantly higher than that of the Ti- and icariine-treated group (*P* < 0.05). Moreover, the RANK expression level was significantly higher in both of them when compared with that of the control (*P* < 0.05, Fig. 7).

### The mRNA level of TRAP, MMP-9, CAII and CtsK in osteoclasts

Real-time PCR results demonstrated that gene expression of TRAP, carbonic anhydrase (CAII), MMP-9 and CtsK were all significantly increased after treatment with Ti or Ti with icariine (*P* < 0.01). In addition, these gene levels were all higher in Ti-treated group with *P* < 0.01 (Table 3).

**Table 3.** The mRNA level of MMP-9, CAII and CtsK in osteoclasts

	Control	Ti	**Ti + icariine**
MMP-9	1*	1.428 ± 0.006**	1.305 ± 0.019*^,^**
CAII	1*	1.577 ± 0.015**	1.273 ± 0.011*^,^**
CtsK	1*	1.523 ± 0.021**	1.382 ± 0.029*^,^**

*P < 0.01compared with Ti.

**P < 0.01 compared with control.

### The protein expression level of RANK, MMP-9 and CtsK in osteoclasts

**Figure 8:**
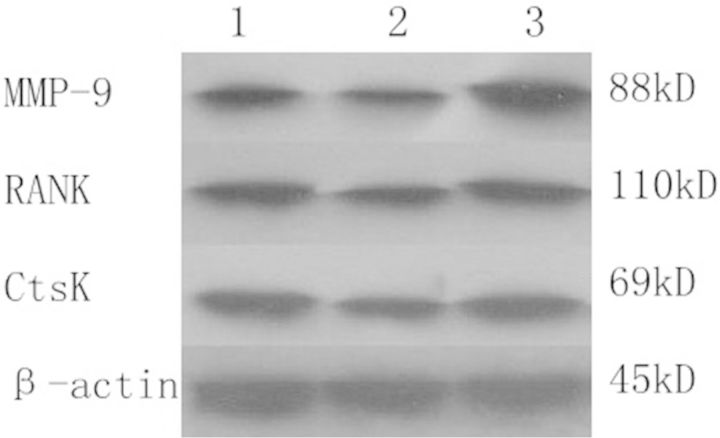
Results from western blot.

Western blot results showed that the expression levels of RANK, MMP-9 and CtsK in osteoclasts were significantly increased after treatment with Ti or Ti with icariine (*P* < 0.05). Moreover, the protein of RANK, MMP-9 and CtsK by Ti treatment was significantly higher than that of the Ti- and icariine-treated group (*P* < 0.05) (Fig. 8, Table 4).

**Table 4:** The protein expression level of RANK, MMP-9 and CtsK in osteoclasts

	Control	Ti	Ti + icariine
MMP-9	1.052 ± 0.028*	1.406 ± 0.017**	1.235 ± 0.012*^,^**
RANK	1.106 ± 0.015*	1.328 ± 0.008**	1.262 ± 0.013*^,^**
CtsK	0.993 ± 0.025*	1.335 ± 0.011**	1.139 ± 0.016*^,^**

*P < 0.01 compared with Ti.

**P < 0.01 compared with control.

## Discussion

After joint replacement, the prosthesis components such as metal, polyethylene, bone cement and surface coating could produce wear debris due to the corrosion and collision of the materials. Wear debris could migrate into the interface between the bone and the implant components, which could induce macrophages to generate inflammation factors, superoxide, metastasis suppressors and metalloproteases [15]. This could in turn induce an intracellular cascade to stimulate cell proliferation, osteoclastogenesis and bone resorption by macrophages and osteoclasts [16]. Wear debris could also improve the proliferation of osteoclasts and osteolysis [17]. Meanwhile, they could inhibit the proliferation and differentiation of osteoblasts, which could disturb the dynamic homeostasis between the bone-forming osteoblasts (bone formation) and the bone-eroding osteoclasts (bone resorption), leading to bone deformity and osteoporosis [18, 19]. Recent studies have shown that the wear debris inhibited the OPG expression, while induced the RANKL/RANK expression, which altered the osteoclastogenesis-dependent RANKL/RANK/OPG system. Thereafter, macrophages were induced to generate the osteoclasts progenitor cells (OPCs), which were matured to osteoclasts. The osteoclasts number increase caused periprosthetic osteolysis and bone resorption, leading to aseptic loosening [20, 21].

The RANKL/RANK/*OPG* system has been shown to play an important role in osteoclasts differentiation [22, 23]. RANKL is mainly produced in periprosthetic osteoblasts and bone marrow stromal cells [24], while RANK is produced by osteoclasts progenitor or osteoclasts cells. The wear debris co-cultured with *OPC*s could lead to a high expression of RANK in the OPCs [25]. The interaction between RANK and RANKL stimulates the osteoclasts precursor to mature into fully differentiated, bone-resorbing osteoclasts, leading to the expression of TRAP and MMP-9. The CAII and CtsK genes are specifically expressed during the substrate decomposing process, mediated by osteoclasts [26, 27]. In this study, the RAW264.7 cells were induced with RANKL for 6 days. The differentiation of osteoclasts from the RAW264.7 cells was improved by Ti treatment. The expression of TRAP, MMP-9, CAII and CtsK were increased. These results indicated that the improvement of osteoclasts differentiation by Ti might have been mediated through the RANKL/RANK system.

Epimedium is an herbaceous perennial, growing from an underground rhizome. It has an acrid-sweet flavor, warm property and effective hepatorenal properties. It is commonly used in Chinese herbal medicine to enhance the erectile function, increase general health and induce anti-rheumatism effects. The primary active constituent in epimedium is icariine, epimedium polysaccharide and flavonoids. Previous studies demonstrated that icariine increased the gene expression level of OPG and induced the osteoblasts proliferation and differentiation, which led to bone formation [28]. Moreover, icariine improved the transforming growth factor, inhibited the expression of TNF-α and RANK-RANKL, which could increase osteoclastogenesis, inhibit bone resorption and increase Ca^2+^ level in the osteoblasts cells. This could result in retraction of actin, reduction of superoxide anion radicals and reduction of the bone resorption pit area [29–31]. Recently, it was reported that icariine has inhibitory effect on inflammatory osteoclastogenesis induced by Ti micro-particles [32]. However, only the optimum order-of-magnitude of icariine concentration was investigated, while the best concentration was not specified [32]. In our study, the mature of RAW264.7 and the bone resorption induced by RANKL were decreased by icariine treatment, and the exact best concentration of the icariine has been found out. In addition, both the number and the area of the bone resorption pits were reduced, the expression level of OPG and RANK was reduced, as well as the mRNA and expression level of TRAP, MMP-9, CAII and CtsK. These results indicated the inhibitory effects of icariine on osteoclasts differentiation, and bone resorption might have been mediated through the RANKL/RANK system.

In summary, osteolysis induced by wear debris appeared to play critical roles in the long-term failure of total joint replacement. How to block this process has become an active research emphasis to protect the aseptic loosening. Our study demonstrated the inhibitory effect of icariine on osteolysis induced by wear debris. However, this was a short-term result from an *in vitro* study. To investigate the long-term effect of osteolysis surrounding the hip prosthesis and to unravel the detailed mechanism of RANKL/RANK system, further studies will be needed.

## Funding

This work was supported by grants from Natural Science Foundation of China (81302986), Young Foundation of Fujian Province Health Department, China (no. 2007-2-37) and Bureau of Science and Technology, Fuzhou (no. 2009-G-104).

*Conflict of interest statement*. None declared.
